# Ceramic Stereolithography of Bioactive Glasses: Influence of Resin Composition on Curing Behavior and Green Body Properties

**DOI:** 10.3390/biomedicines10020395

**Published:** 2022-02-07

**Authors:** Qirong Chen, Franziska Schmidt, Oliver Görke, Anila Asif, Joachim Weinhold, Erfan Aghaei, Ihtesham ur Rehman, Aleksander Gurlo, Asma Tufail Shah

**Affiliations:** 1 Technische Universität Berlin, Faculty III Process Sciences, Institute of Materials Science and Technology, Fachgebiet Keramische Werkstoffe/Chair of Advanced Ceramic Materials, Straße des 17. Juni 135, 10623 Berlin, Germany; chenqir0923@gmail.com (Q.C.); aghaeierfan90@yahoo.com (E.A.); 2 Charité—Universitätsmedizin Berlin, Corporate Member of Freie Universität Berlin and Humboldt Universität zu Berlin, Department of Prosthodontics, Geriatric Dentistry and Craniomandibular Disorders, Aßmannshauser Str. 4–6, 14197 Berlin, Germany; franziska.schmidt2@charite.de; 3 Interdisciplinary Research Centre in Biomedical Materials, COMSATS University Islamabad Lahore Campus, Defence Road, Off-Raiwand Road, Lahore 54000, Pakistan; anilaasif@cuilahore.edu.pk; 4 Technische Universität Berlin, Faculty II—Mathematics and Natural Sciences, Institute of Mathematics, 3D Lab, Straße des 17. Juni 135, 10623 Berlin, Germany; weinhold@math.tu-berlin.de; 5 Engineering Department, Lancaster University, Gillow Ave, Bailrigg, Lancaster LA1 4YR, UK; i.u.rehman@lancaster.ac.uk

**Keywords:** additive manufacturing, resin development, ceramic stereolithography, bioactive glass, porous scaffolds

## Abstract

Herein we report on the preparation of a bioactive glass (BAG)-based photocurable resin for the additive manufacturing of BAG scaffolds with high filler loadings. The preparation of glass/ceramics resins for stereolithography with high filler loading is always a challenge, especially for fillers with a high refractive index variance. Various photocurable resin compositions with and without bioactive glass fillers have been investigated to see the influence of bioactive glass on physical properties of the resin and resulting green body. The effect of concentration of monomers, reactive diluent, light absorber (Sudan orange G dye), photoinitiator (PI), non-reactive diluent, and fillers (BAG) on rheology and photocuring behavior of the resin and tomography of the resulting 3D structures have been investigated. The BAG contents affect the rheology of resin and influence the rate of the polymerization reaction. The resin compositions with 55–60% BAG, 10% PEG-200 (diluent), 1% of PI and 0.015% of the dye were found to be suitable compositions for the stereolithographic fabrication. A higher percentage of PI caused over-curing, while a higher amount of dye decreased the cure depth of the resin. The micro-computed tomography (µ-CT) and scanning electron microscopic (SEM) images of the resulting green bodies display a relatively dense glass scaffold without any visible cracks and good interlayer connection and surface finishing. These properties play an important role in the mechanical behavior of 3D scaffolds. This study will be helpful to prepare high density glass/ceramic slurries and optimize their printing properties.

## 1. Introduction

Bioactive glass (BAG) is a biomaterial with the ability to form a chemical bond with bone through the formation of carbonated hydroxyapatite [1]. It has been extensively used as bone and dental filler in biomedical applications due to its excellent biocompatibility, bioactivity, and osteoconductivity [2]. The use of BAG at load-bearing sites is unfortunately restricted by its inherent brittleness and poor mechanical strength, in bulk and especially in the form of scaffolds [3]. The mechanical and physical properties of BAG scaffolds can be enhanced by controlling their geometry, such as pore sizes, pore distribution and strut geometry. Additive manufacturing (AM) allows developing of such scaffolds with controlled geometry at the nano- and micro-metric scale [4,5]. The porous structure with a well-defined geometry is developed by computer-aided design (CAD) and subsequently produced by AM through layer-by-layer deposition [6]. Different AM processes are used for the fabrication of scaffolds including stereolithography (SLA) [7], material jetting, binder jetting, material extrusion, powder bed fusion, sheet lamination, and directed energy deposition [8]. SLA is one of the oldest AM techniques where 3D structures with controlled architecture and resolution are fabricated by light-induced crosslinking of photocurable liquid polymer-based resins [9]. Thus far, most investigations focus on the development of epoxy-based blends, such as by Tosto et al. [10] or the influence of dual curing, e.g., photocuring followed by thermal curing to enhance mechanical strength of the green body [11]. However, AM of glass/ceramics by SLA is still a challenge, mostly due to the high refractive index difference between glass/ceramic particles and photoreactive polymers [12]. Polymer-based routes employing preceramic polymers without filler particles have been developed as an alternative to fabricate silicate ceramics by SLA for SiOC and SiC ceramics [13]. However, for compositions outside of that range, a high solid loading of glass/ceramic particles inside the resin with homogeneous distribution is necessary for the fabrication of high-density scaffolds using SLA. The type and volume fraction of the filler, as well as the particle size distribution, strongly influence the viscosity and curing behavior of the filled resin [8]. Depending on particle size and the refractive index of both the ceramic and photoreactive polymer, the curing depth may be reduced more or less drastically [14], and a broadening effect on the curing spot can occur, decreasing the resolution of the printed structure and increasing the overcuring effect and inaccuracy of the printing process [15]. Previous studies reported that a ceramic filler content up to 40 vol. % is limited to materials with micro-sized particles [16]. Poor dispersion and sedimentation issues cause an inhomogeneous distribution of the filler particles inside the printed structure. Most of the present studies on ceramic SLA are focused on the fabrication of SiO_2_, Al_2_O_3_ and its ceramic derivatives. There are very few reports on fabrication of 3D printed bioactive glass by SLA [5,17]. However, none of these have discussed the detailed investigation of the components on resin properties and photo-curing. The main focus of most of the previous studies was to evaluate the influence of the fabrication as well as debinding parameters on the properties of the resulting BAG components.

In this manuscript, acrylate-based photocurable resin compositions without and with BAG particles (d50 = 4 µm) were prepared. To optimize the properties of the resin regarding processability, mechanical stability and shelf life, the concentrations of monomers, a reactive diluent, photoinitiator/co-initiator (PI), non-reactive diluent/dispersant and light absorber were considered and the influence of each component on resin rheology, cure depth during the stereolithography process as well as the hardness of the fabricated parts was studied in detail. The curing conditions, such as the light intensity and exposure time, have been investigated to ensure the effective polymerization of the ceramic layers while minimizing the scattering effect of ceramic particles. These complicated factors limit the wider use of lithography-based AM techniques in the production of porous ceramic scaffolds with advanced functions. Finally, the effect of the ceramic resin composition on the structure of printed green bodies was investigated using micro-computed tomography (micro-CT). This study advances the development of a stable glass/ceramic resin composition suitable for ceramic stereolithography and provides a detailed understanding of the influence of the resin components on the cure depth and physical properties of scaffolds.

## 2. Materials and Methods

### 2.1. Materials

Bioactive glass powder with a mean diameter of 4 μm (d50 = 4 ± 1 μm) and a specific surface area of 1.5 m^2^/g was purchased from Schott (Mainz, Germany). Trimethylolpropane ethoxylate triacrylate (TMPE-OTA, Sigma-Aldrich, St. Louis, MO, USA), 2-hydroxyethyl acrylate (HEA, Sigma-Aldrich, St. Louis, MO, USA; 96%), camphorquinone (CQ, Sigma-Aldrich, St. Louis, MO, USA; used as a photoinitiator), 4-(dimethylamino) benzaldehyde (DMAB; Sigma-Aldrich, St. Louis, MO, USA; co-initiator), Sudan Orange G dye (Sigma-Aldrich, St. Louis, MO, USA, used as a light absorber) and polyethyleneglycol-200 (PEG-200, Merck, Darmstadt, Germany) were used in analytical grade without further purification. CQ is a type II photoinitiator which functions by intramolecular abstraction from tertiary amines [18]. It absorbs light at a wavelength longer than 400 nm and is applicable for the visible light region. A combination of CQ with co-initiator DMAB has been reported to be biocompatible and is used in many dental resins [19]. TMPE-OTA was used as a base monomer due to its low viscosity and high reactivity, while HEA was employed as a reactive diluent. The physical properties of the materials applied in this work are given in Table S1.

### 2.2. Preparation of Photocurable Resins 

The photocurable resin was prepared using monomers TMPE-OTA and HEA in various ratios, CQ (photo-initiator) and DMAB (co-initiator) with a CQ:DMAB weight ratio = 1:1, Sudan Orange G dye (as a light absorber) and PEG-200 (as a non-reactive diluent and rheology modifier). Various loadings of BAG filler (20–65 wt.%) were added into the resin. The final mixture was homogenized in a milling vessel using a roller bank at a speed of 50 min^−1^ for 18 h of milling. An overview of different resin compositions investigated in this study is given in Table 1 and Table 2. The resin composition was described as B_a_M_b_PI_c_D_d_E_e_, where the letters B, M, PI, D and E represent the BAG, monomers, photo-initiator, dye and diluent (PEG-200), respectively. The small letters in subscript, i.e., a, b, c, d and e show the concentration/ weight fraction of these components as explained below:

B**_a_** = weight fraction of BAG with respect to acrylate monomers (TMPE-OTA and HEA), i.e., m_BAG_/m_acr_ + m_BAG_, [%].

M**_b_** = weight ratio of TMPE-OTA in the monomer’s mixture (TMPE-OTA and HEA) m_acr_ = m_TMPE_ + m_HEA_, for example, M_90_ consists of 90 wt.% TMPE-OTA and 10 wt.% HEA.

PI**_c_** = weight fraction of the photo-initiator with respect to total acrylate monomers weight, i.e., c= m_PI_/(m_TMPE_ + m_HEA_), [%].

D**_d_** = weight fraction of the dye (Sudan Orange G) with respect to total acrylate monomers weight, i.e., d= m_dye_/(m_TMPE_ + m_HEA_), [%].

E**_e_** = weight fraction of PEG-200, where e = m_PEG_/(m_acr_ + m_PEG_), [%], while m_BAG_ + m_acr_ + m_PEG_ is considered as 100 percent; for details, see Table 1 and Table 2, and Tables S2 and S3 in supplementary information.

### 2.3. Fabrication of BAG Scaffolds

A Lithoz CeraFab 7500 (Lithoz GmbH, 1060 Vienna, Austria) system was used for the photopolymerization. The wavelength range of the light source was between 400 and 500 nm. The thickness of the layer of the printed scaffold was set to 25 µm and lateral resolution reached 40 µm. The light exposure time of the first five layers was 3900 ms and for the following layers it was 3500 ms.

**Figure 1:**
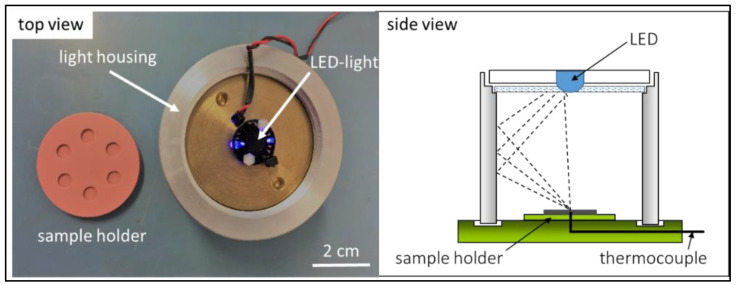
The in-house developed set-up used for curing experiments of resins without and with bioactive glass fillers. The top view (**left side**) is a photographic image of the sample holder (made from silicone) and the light source (LED) for curing tests. The side view (**right side**) gives a schematic of the equipment. The sample holder is located in the middle bottom, while light is emitted from the LED at the top of the set-up.

However, the initial curing reactions for optimization of the concentration of monomers, PI, dye and PEG-200 were carried out using an in-house developed set-up shown in Figure 1. The setup consisted of a hollow metallic cylinder with an inner diameter of 72 mm and with an LED (3 W, 460–470 nm) located on the top of the cylinder. The light exposure time was varied between 10–180 s. The cure depth was measured by a Vernier caliper (n = 5) after washing the cured samples with isopropanol to remove the uncured monomers [20]. 

### 2.4. Characterization Techniques

The particle size distribution of the BAG was analyzed on a Beckman Coulter LS 13 320 laser diffraction particle size analyzer (Brea, MA, USA) in Universal Liquid Mode (ULM). The powders were dispersed in water using a probe sonicator to obtain a homogeneous distribution of particles in the water and avoid agglomeration. The particle size measurement was performed in terms of volume percentage (vol.%).

The rheology was determined by using a Physica MCR 301 Rheometer (Anton Paar, Graz, Austria). The PP 25 parallel plate geometry (Ø = 25 mm) was used to measure the rheological behavior of each polymer and resin compositions. The gap between resin and plate was set to 0.5 mm, with the shearing rate increasing linearly from 1 to 200 s^−1^ or 600 s^−1^ at a ramp rate of 1 s^−1^.

Photo-Differential Scanning Calorimetry (DSC) under exposure to UV light in the range between 250 and 600 nm at 10, 50 and 100 mW/s was conducted to measure heat flow during curing. The measurements were performed by using a DSC Q2000 (TA Instruments, Milford, MA, USA) calorimeter.

Imaging of the green body was performed by Scanning Electron Microscopy (SEM) employing a Gemini LEO 1530 FEG-SEM (Carl Zeiss AG, Oberkochen, Germany) with a Noran EDX system by Thermo Fisher Scientific. 

Structural analysis was carried out using micro-computed tomography (µCT) scanning on a Phoenix Nanotom M (General Electrics, Boston, MA, USA) at a voxel size of 14.99 µm. The scan was carried out at a voltage of 140 kV, a current of 160 µA, using large tube mode (0) and without the usage of filters.

## 3. Results and Discussion

### 3.1. Selection of the Acrylate Composition and Dye Content in the Resin

Photocurable resins comprise reactive monomers (in our experiment two different acrylates monomers TMPE-OTA and HEA), PI (CQ—photoinitiator and DMAB—co-initiator) and a light absorber (Sudan orange G dye). To understand the role of each component in the resin different resin, the compositions M_b_PI_c_D_d_ without bioactive glass filler were prepared and investigated.

**Figure 2:**
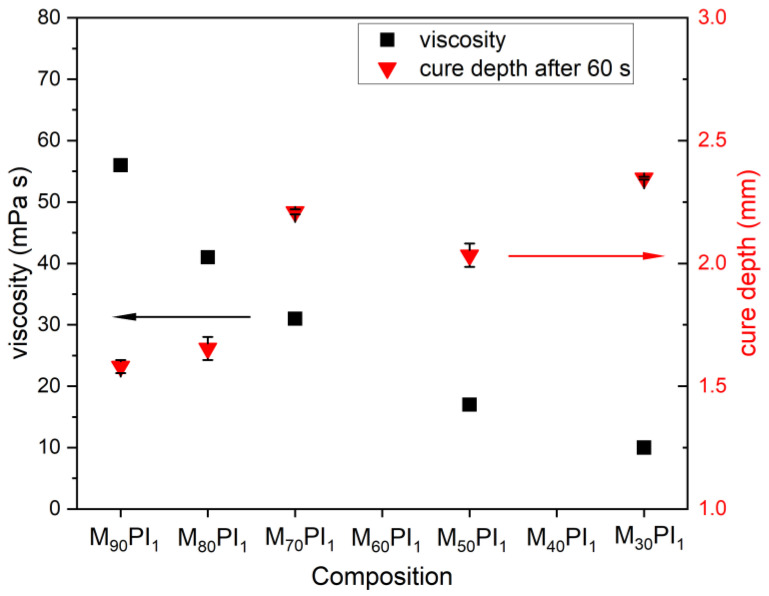
Influence of monomer content (ratio of TMPE-OTA and HEA) on resin properties. The left-side y-axis shows viscosity values (black squares ■, at shear rate 200 s^−1^) and the right-side y-axis shows the achieved curing depth after 60 s of light exposure (red triangles ▼) (error bars show the mean variation).

In the first step, the influence of the acrylate composition (meaning ratio of TMPE-OTA and HEA monomers) on resin viscosity and curing depth of the resin was investigated (Figure 2). It has been observed that with increasing HEA content in M_b_PI_c_ (without dye and bioactive glass) samples, the viscosity of the resin (Figure 2, viscosity measured at shear rate 200 s^−1^) rapidly decreased. This is due to HEA being a smaller monomer with a lower density and initial viscosity compared to TMPE-OTA (Table S1). The lowest viscosity observed was 10 mPa.s for M_30_PI_1_, while for M_90_PI_1_ with the highest HEA content, the viscosity was 56 mPa.s. The monomer contents also had a significant influence on the photocuring behavior of the specimens. Figure 2 also shows that the value of cure depth increases with an increase in the HEA content in resin up to M_70_PI_1_(30% HEA), while a further increase led to gel-like behavior for both M_30_PI_1_ and M_50_PI_1_ due to a higher content of less viscous HEA and a low percentage of the triacrylate TMPE-OTA.

**Figure 3:**
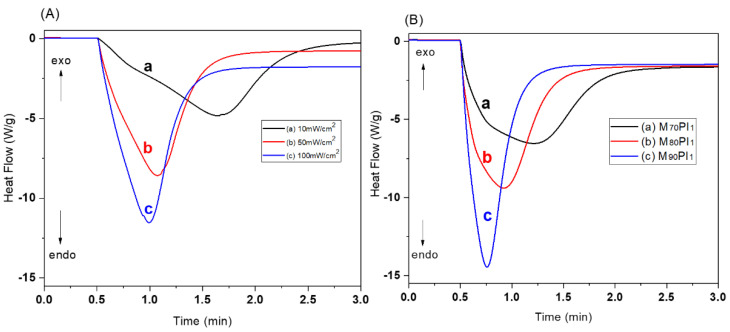
(**A**) Influence of irradiation power of (**a**) 10, (**b**) 50, and (**c**) 100 mW/cm^2^ on the heat flow (photopolymerization) of sample M_80_PI_1_D_0.005_ measured by in situ UV-DSC. (**B**) Influence of resin composition at an irradiation power of 50 mW/cm² on heat flow profiles (photopolymerization) for resins M_70_PI_1_ (**a**), M_80_PI_1_ (**b**) and M_90_PI_1_ (**c**) measured by UV-DSC.

The resins M_90_PI_1_, M_80_PI_1_ and M_70_PI_1_ were selected for further investigations, because other compositions showed semi-solid/gel-like behavior during the curing as discussed above. In situ UV-DSC was used to understand the effect of light intensity and HEA content on photopolymerization of acrylates. Three different intensities of UV light, i.e., 10, 50 and 100 mW/cm^2^ (Figure 3A), were used for in situ photo-curing of the sample M_80_PI_1_D_0.01_, to determine the effect of light intensity as a function of time on photopolymerization of acrylates. The amount of exothermic heat tends to increase as the light intensity increases as shown in Figure 3A [21]. The time that the resin reaches the exothermic peak represents the earlier starting point. Figure 3A shows the high reaction speed at 10 mW/cm^2^ intensity reaches maxima at 1.6 min, while at 50 mW/cm^2^, it took 1.0 min. The reaction induction time reduces during the curing at higher light intensity depicting stronger exothermic reaction, which may entrap many unreacted radicals. Therefore, for further investigations, 50 mW/cm^2^ light intensity was used for the polymerization reactions. Figure 3B shows the time-dependent heat release profiles for several resin compositions at 50 mW/cm². The amount of heat released, as determined by the area beneath the peak, for samples M_70_PI_1_, M_80_PI_1_, and M_90_PI_1_ (with increasing HEA contents), were 357.4, 322.3, and 313.2 J/g, respectively. Thus, the amount of energy released was almost proportional to the HEA contents. In contrast, the rate of polymerization decreased with an increase of HEA ratio. As the TMPE-OTA molecule possesses three acrylate groups, a higher TMPE-OTA content resulted in accelerated photopolymerization and higher heat flow in M_90_PI_1_ (15 W/g, 0.75 min) as compared to M_70_PI_1_ (7 W/g, 1.25 min) [22].

**Figure 4:**
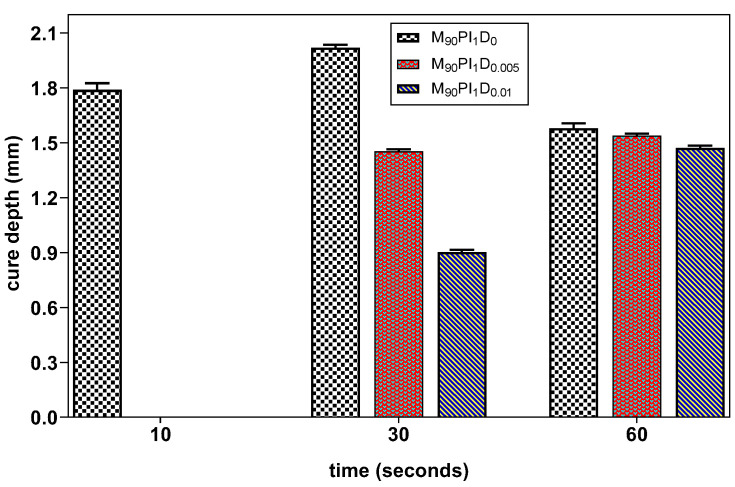
Influence of the dye (Sudan orange G dye) content on the cure depth of resins M_90_PI_1_D_0_, M_90_PI_1_D_0.005_ and M_90_PI_1_D_0.01_ (error bars show the mean variation).

Dye in photocurable resins is used to absorb photons (without creating free radicals) and suppress the light scattering that may occur due to deflection by filler particles over the slurry layers, which helps to control the overgrowth [23]. Therefore, the influence of the dye content on the photocuring behavior of the resin was investigated for a more reactive acrylate composition M_90_PI_1_. Figure 4 shows that the dye content has an influence on the cure depth of the resin at 10 and 30 s exposure time. In M_90_PI_1_D_0_, curing took place after 10 s exposure, while both M_90_PI_1_D_0.005_ and M_90_PI_1_D_0.01_ remained in gel form at this exposure time. On increasing the exposure time to 30 s, the cure depth of M_90_PI_1_D_0_, M_90_PI_1_D_0.005_ and M_90_PI_1_D_0.01_ was 2.02, 1.45 and 0.45 mm, respectively. Hence, samples with more dye content showed less curing depth. When the exposure time increases to 60 s, curing volume shrinkage was observed in the M_90_PI_1_D_0_ (sample without dye). For both M_90_PI_1_D_0.005_ and M_90_PI_1_D_0.01_ samples with different dye contents, the curing depth value increases at 60 s exposure time because the available free radicals which may cause volume shrinkage are readily absorbed by dye.

Based on above mentioned results, in the first instance—based on the higher photopolymerization rates (Figure 3B)—the compositions M_80_PI_1_ and M_90_PI_1_ with a higher TMPE-OTA content were selected for the formulation of resins with BAG particles. In addition, though the dye content showed no direct effect on photopolymerization of the monomers, it clearly reduced the overcuring. Therefore, the quantity of dye in the following experiments was related to the mass fraction of BAG, i.e., the higher the percentage of BAG, the higher the amount of dye required to control the light scattering.

### 3.2. Optimization of Ceramic Resin Compositions

**Figure 5:**
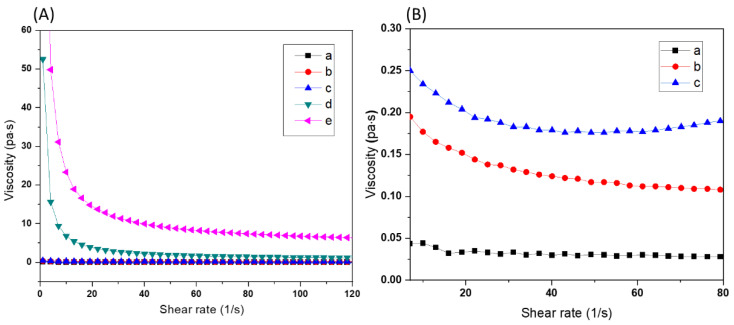
(**A**) Influence of bioactive glass content on the viscosity of bioactive glass containing resins (**a**) 20BM_90_PI_1_D_0.005_, (**b**) 30BM_90_PI_1_D_0.005_, (**c**) 40BM_90_PI_1_D_0.005_, (**d**) 50BM_90_PI_1_D_0.005_ and (**e**) 60BM_90_PI_1_D_0.005_. (**B**) The non-Newtonian behavior of samples 20BM_90_PI_1_D_0.005_ (**a**), 30BM_90_PI_1_D_0.005_ (**b**) and 40BM_90_PI_1_D_0.005_ (**c**) at low shear rates.

To investigate the influence of the BAG content on the viscosity of the ceramic resins, xBM_90_PI_1_D_0.005_ resins with 1% PI, 0.005% dye and different BAG contents, ranging from 20 to 60% (m/m_resin_), were compared (Table 2). PEG-200 was not incorporated in any of these resins. The viscosity of resin compositions 20BM_90_PI_1_D_0.005_, 30BM_90_PI_1_D_0.005_ and 40BM_90_PI_1_D_0.005_ was very low (Figure 5A).The resin 20BM_90_PI_1_D_0.005_ showed non-Newtonian viscosity (Figure 5B) at low shear rates due to inhomogeneous dispersion at lower BAG contents, while both 30BM_90_PI_1_D_0.005_ and 40BM_90_PI_1_D_0.005_ resins showed shear thinning behavior at shear rates below 30 s^−1^. At higher BAG contents, the viscosity of 50BM_90_PI_1_D_0.005_ and 60BM_90_PI_1_D_0.005_ was very high (up to 50 Pa.s) at low shear rates but decreased with an increase in shear rate. It is a typical shear thinning behavior (pseudoplastic) of glass/ceramic inks.

In the next step, compositions with a higher BAG content, i.e., 45–60% were selected for further optimization (i.e., stability, hardness, and rate of photopolymerization) of the photocurable resin.

**Figure 6:**
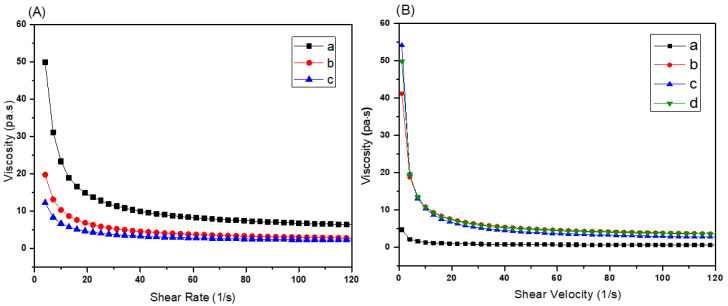
(**A**) Influence of PEG-200 on viscosity (**a**) 60BM_90_PI_1_D_0.005_E_0_, (**b**) 60BM_90_PI_1_D_0.005_E_10_ and (**c**) 60BM_90_PI_1_D_0.005_E_15_. (**B**) Influence of bioactive glass content on the viscosity of the resin (**a**) 45BM_90_PI_1_D_0.005_E_10_, (**b**) 55BM_90_PI_1_D_0.005_E_10_, (**c**) 60BM_90_PI_1_D_0.005_E_10_ and (**d**) 65BM_90_PI_1_D_0.005_E_10_.

To improve the rheological behavior of BAG containing resins, PEG-200 was employed. PEG has been reported to improve resin flowability while being non-reactive [24]. Figure 6A shows the rheology of resins 60BM_90_PI_1_D_0.005_E_e_ with 60% m/m_resin_ BAG and varying amounts (i.e., 0, 10 and 15%) of PEG-200. The viscosity of the resin 60BM_90_PI_1_D_0.005_E_0_ was 14.9 Pa.s at a shear rate of 20 s^−1^, while on addition of 10% PEG-200, the viscosity value decreased to 6.6 Pa.s. On further increasing the PEG-200 contents to 15%, the viscosity value decreased to 4.2 Pa.s. However, the difference in viscosity for 10 and 15% PEG-200 was small. Therefore, 10% mass fraction of PEG-200 was selected for further experiments. Figure 6B shows the influence of BAG contents on the rheology of xBM_90_PI_1_D_0.005_E_10_ with 10% PEG-200. In presence of PEG-200, the rheology was almost independent of the amount of BAG (55% to 65% mass fraction) [23], which allows the application of these bioactive glass resins with high filler content in stereolithography.

**Figure 7:**
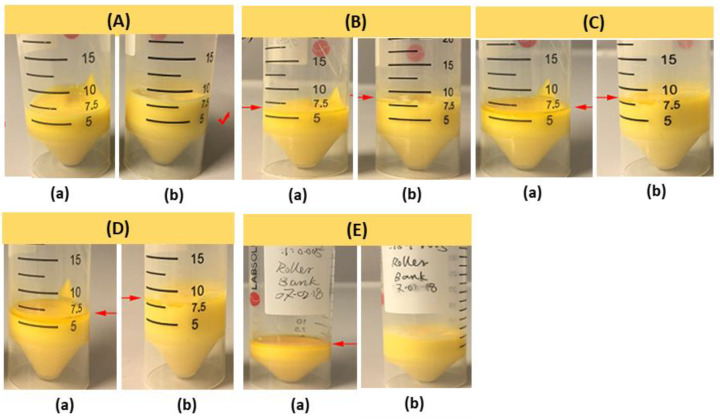
Photographic images showing the shelf life of resin 55BM_80_PI_1_D_0.005_ with 55% bioactive glass determined at room temperature for different time periods; (**A**) day 1, (**B**) day 2, (**C**) day 6, (**D**) day 10 and (**E**) day 43. The falcon tubes (**a**) are without PEG-200 (55BM_80_PI_1_D_0.005_E_0_), while faclcon tubes (**b**) contains 10% PEG-200 (55BM_80_PI_1_D_0.005_E_10_).

PEG-200 also plays a role in the shelf life and stability of the resin formulation. The shelf life of BAG resin 55BM_80_PI_1_D_0.005_E_10_ was studied at different time intervals for a period of 43 days at room temperature. The results are shown in Figure 7. Resin compositions 55BM_80_PI_1_D_0.005_E_0_ started to settle on day 2, as can be seen in Figure 7B, while resin composition 55BM_80_PI_1_D_0.005_E_10_ was stable even after day 43. Hence, PEG-200 improved the stability and shelf life of the resin. A stable and homogeneous suspension leads to the homogeneity of the microstructure of the sintered parts and improved mechanical strength. 

The effect of the amount of PEG-200 on the shore-D hardness of samples (curing time = 60 s) was also investigated (Table 3). The shore-D measurements showed that the hardness value decreases with increasing amounts of PEG-200. This is because PEG-200 acts as a plasticizer and is also known to lowers the Young’s modulus [25]. However, when increasing the weight fraction of PEG-200 from 10 to 15%, a very small change in rheology was observed, as discussed above. Henceforth, a 10% weight fraction of PEG-200 was chosen for the additive manufacturing of BAG resin compositions in order to avoid any change in mechanical properties.

**Figure 8:**
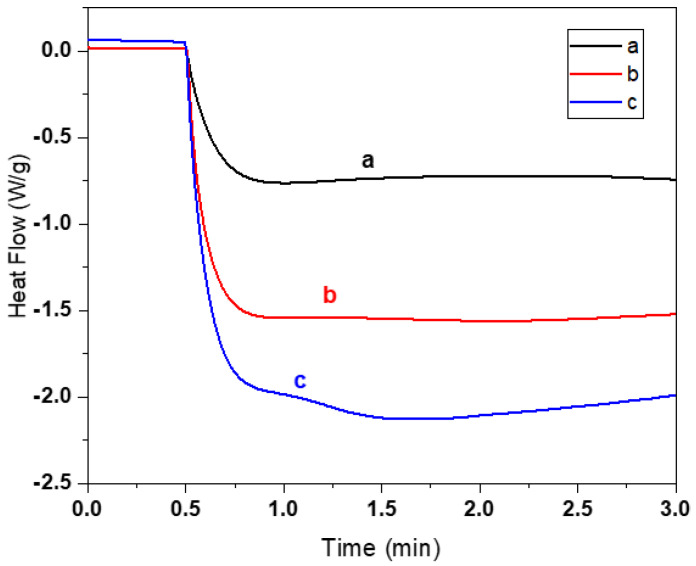
Influence of BAG contents on the heat flow (indicator of photopolymerization) of resin xBM_80_PI_1_D_0.01_E_10_ irradiated with 50 mW/cm² (**a**) 60BM_80_PI_1_D_0.01_E_10_ with 60% BAG, (**b**) 55BM_80_PI_1_D_0.01_E_10_ with 55% BAG and (**c**) 40BM_80_PI_1_D_0.01_E_10_ with 40% BAG.

Furthermore, the influence of the BAG content on the photopolymerization reaction was investigated by in situ UV-DSC. Figure 8 shows the effect of different amounts of BAG on the photopolymerization reaction of photocurable resins with 1% PI and 0.01% dye (both m/m_acr_) and 10% PEG-200 (m/m_resin_). The thermogram indicates that the photopolymerization reaction for resin compositions with BAG was unfinished even after 3 min of measurement. These BAG resin compositions xBM_80_PI_1_D_0.01_E_10_ contain a high mass fraction of triacrylate (80TMPE-OTA:20HEA), and therefore, these mixtures would tend to trap more radicals and more unreacted monomer. Another factor might be the scattering effect and absorption of light by BAG particles due to the difference in refractive index value of monomers and fillers. The refractive index of BAG (bioglass 45S5) is 1.54–1.56 [21], while the refractive index of acrylates is 1.44–1.47. Figure 8 also shows that there was a difference in heat profile in relation to the BAG content in the resins. At high BAG loading, less heat was released per unit mass. An increase in weight fraction of BAG per unit mass in the composition leads to a relative decrease in the number of monomers and, consequently, less heat is released per gram. In the case of 40BM_80_PI_1_D_0.01_E_10_, two endothermic dips (fluctuations) were observed, one after 0.8 min and the other at 1.5 min, which could be related to inhomogeneity of the resin with lower BAG contents. Hence, BAG contents not only affect the rheology of the resin but also strongly influence the rate of the polymerization reaction.

**Figure 9:**
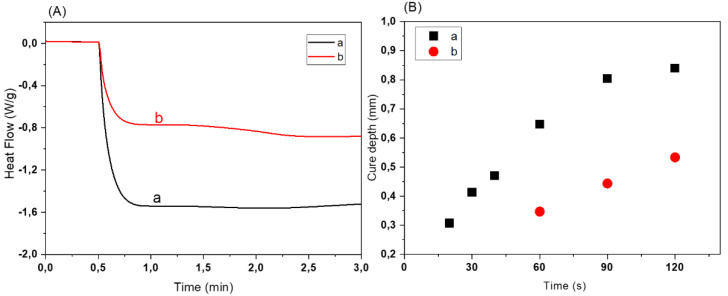
(**A**) Influence of dye on heat flow (indicator for photopolymerization) for resin 50BM_90_PI_1_D_d_ with 50% m/m_resin_ bioactive glass contents (**a**) 50BM_90_PI_1_D_0_ without dye and (**b**) 50BM_90_PI_1_D_0.01_ with 0.01% dye. (**B**) Influence of dye contents on cure depth of the resin (**a**) 50BM_90_PI_1_D_0.005_ (black square ■) with 0.005% dye and (**b**) 50BM_90_PI_1_D_0.01_ (red circle ●) with 0.01% dye.

As discussed above, BAG particles scatter light due to a refractive index contrast with the monomer and, consequently, the cure depth is affected, which can cause overgrowth or uncontrolled curing [26]. The dye has been reported to suppress light scattering (due to deflection by filler particles) and control the overgrowth as mentioned earlier [27]. In situ UV-DSC measurements of BAG resins showed difference in heat released per unit mass for different amounts of the dye in the resin (Figure 9A). For the resin 50BM_90_PI_1_D_0_ without dye (a), the heat released was about two times greater than for resin 50BM_90_PI_1_D_0.01_ (b). Hence, the dye has a direct correlation with the presence of BAG. It absorbs the free radicals and prevents the protuberant scattering effect of BAG particles. This is supported by Figure 9B, where the cure depth of two different resin compositions 50BM_90_PI_1_D_0.005_ and 50BM_90_PI_1_D_0.01_ with various amounts of dye were investigated. The cure depth of sample 50BM_90_PI_1_D_0.005_ was 0.4 mm after 30 s light exposure time, while no curing was observed for 50BM_90_PI_1_D_0.01_. Hence, an increase in amount of dye from 0.005 to 0.01% (m/m_acr_) delayed the curing time.

**Figure 10:**
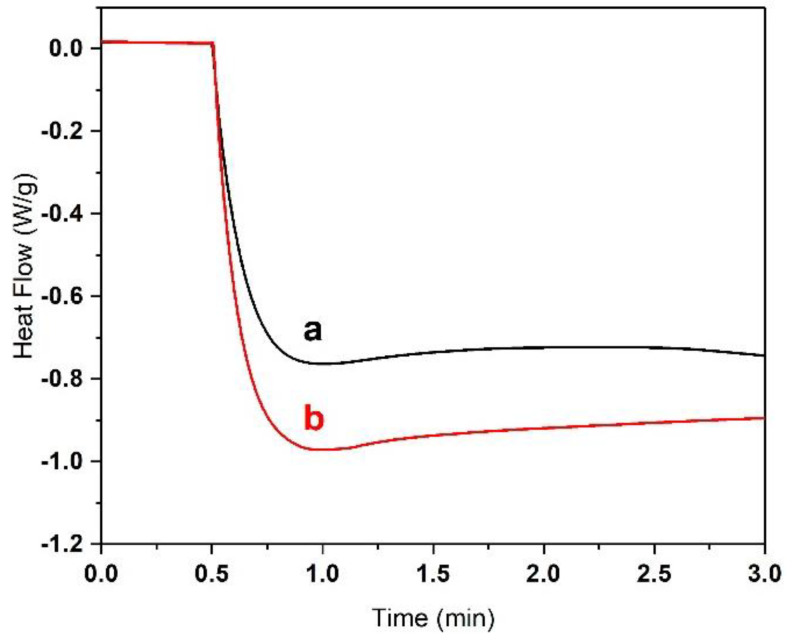
The influence of concentration of photo-initiator on heat flow (indicator of photopolymerization) in the resin, (**a**) 55BM_80_PI_1_D_0.01_E_10_ with 1% (m/m_acr_) PI and (**b**) 55BM_80_PI_2_D_0.01_E_10_ with 2% (m/m_acr_) PI.

In order to investigate the effect of PI on the photopolymerization, two different resin compositions, 55BM_80_PI_1_D_0.01_E_10_ and 55BM_80_PI_2_D_0.01_E_10_, with 1 and 2% (m/m_acr_) PI, respectively, were selected and their effect on photo-curing was investigated by UV-DSC. Figure 10 shows expeditious curing of the resin with higher percentage of PI due to the availability of more free radicals. Nevertheless, the resin composition with 1% m/m_acr_ PI was selected for all photo-curing reactions to avoid rapid conversion, which can cause crack formation.

### 3.3. Fabrication of 3D Scaffolds

Two different ceramic resin compositions, 55BM_80_PI_1_D_0.015_E_10_ and 60BM_80_PI_1_D_0.015_E_10_, were chosen for 3D printing in the Lithoz Ceracet. The layer thickness was set at 25 µm with backlight exposure of 4 s and main light exposure of 4 s for each layer. In order to investigate the effect of BAG loading, first a gear-shaped sample was prepared with two different dimensions. The printing test was completed successfully with an accurate desired structure. Moreover, no scattering effect was observed with these two compositions. On the macro-scale, the parts appeared to be defect-free and the surface was flat and smooth.

**Figure 11:**
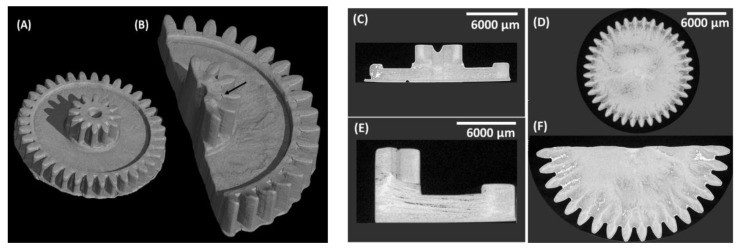
µ-CT images of the gear-shaped objects fabricated from 55BM_80_PI_1_D_0.015_E_10_ (**A**,**C**,**D**) and 60BM_80_PI_1_D_0.015_E_10_ (**B**,**E**,**F**) resins. (**C**,**E**) are side views of (**D**,**F**).

The µ-CT of the printed samples was performed to investigate the detailed structure and homogeneity. As shown in the top view in Figure 11, the printing direction is from bottom to top, which is also expressed by the sequence of µ-CT pictures. The bright area represents the materials and the dark area represents air; the brighter the area, the higher the density of the material. The side view µ-CT images show the layer connections in the printed samples. Comparing the side view images, it appears that the sample 55BM_80_PI_1_D_0.015_E_10_ (small gear-shaped structure) is more uniform and homogeneous as compared to the sample 60BM_80_PI_1_D_0.015_E_10_ (large gear-shaped structure) with higher bioactive glass loading (60% weight fraction BAG). The layers were interdiffused in 55BM_80_PI_1_D_0.015_E_10_; however, some grains appeared during µ-CT, which might be due to aggregates. In contrast in 60BM_80_PI_1_D_0.015_E_10_, the delamination of layers was observed at a few places without the appearance of any aggregation.

**Figure 12:**
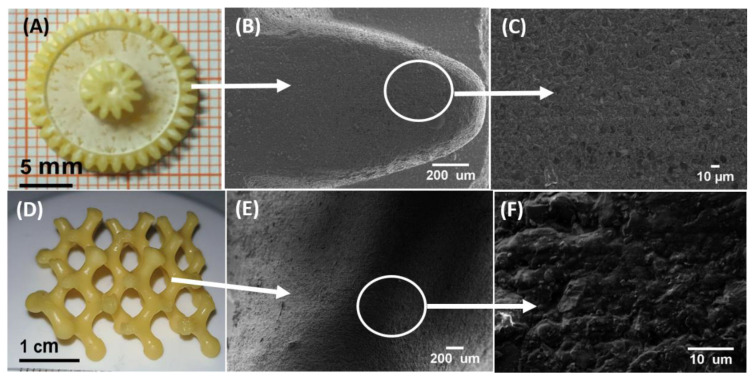
The photographic images of 3D printed small gear-shaped (**A**) and honey-comb (**D**) samples with composition 55BM_80_PI_1_D_0.015_E_10_. (**B**,**C**,**E**,**F**) SEM images of these 3D printed samples.

Figure 12 shows the 3D printed gear-shaped and honey-comb structure with composition 55BM_80_PI_1_D_0.015_E_10_. No visible cracks or layer separation on the surface of the printed structure was seen in SEM images (Figure 12B,E). However, a few lines parallel distributed on the surface of the cross-section were observed (Figure 12C), which can be attributed to the layers’ connection in the printing process. The images at various magnifications showed that the printed samples were crack-free and dense (Supplementary information Figure S1).

SEM and µ-CT results showed that the sample 55BM_80_PI_1_D_0.015_E_10_ was the most optimized BAG resin composition for ceramic stereolithography of bioactive glass scaffolds. No cracks were observed in printed samples and SEM images at high magnification showed that the printed sample has a dense structure.

Henceforth, a suitable resin composition for 3D printing of bioactive glass was achieved by investigating the physical properties, including rheology, heat flow and morphology. These investigations will be helpful to formulate the resin for 3D printing of ceramics/glasses for various applications. 

## 4. Conclusions

Ceramic stereolithography is a very suitable method for the fabrication of implants and scaffolds for tissue engineering with controlled porosity as they can be fabricated according to patient-specific requirements. The results showed that the rate of photopolymerization increases with an increase in the concentration of acrylate monomers due to an increase in the number of unsaturated C=C groups per unit gram of monomer units. Resin compositions with a TMPE-OTA:HEA weight ratio of 90:10 and 80:20 were suitable for BAG printing due to optimal resin viscosity. To increase the flowability of BAG resin, PEG-200 was used as diluent and plasticizer, 10% of PEG-200 was found to be the appropriate amount for the fabrication. At this PEG-200 content, the viscosity of bioactive glass resin remains almost constant irrespective of glass loading in the range from 55 to 65 wt.%. The light scattering effect (due to deflection by filler particles) over the slurry layers can be controlled by using appropriate amounts of light absorbent (dye) which absorb photons (without creating free radicals) and help to control the overgrowth. Two compositions, 55BM_80_PI_1_D_0.015_E_10_ and 60BM_80_PI_1_D_0.015_E_10_, with 55 and 60% BAG, 10% PEG-200, 1% of PI and 0.015% of Sudan orange G dye were found to be the suitable for the stereolithographic fabrication and were successfully used to fabricate uniform scaffolds with different shapes without the appearance of macro-cracks. 

## Figures and Tables

**Table 1:** The compositions of the resins without bioactive glass investigated in this study.

Samples	m_TMPE_, [%]	m_HEA_, [%]	PI, m_PI_/m_acr,_ [%]	Dye, m_dye_/m_acr,_ [%]
M_90_PI_1_	90	10	1	-
M_80_PI_1_	80	20	1	-
M_70_PI_1_	70	30	1	-
M_50_PI_1_	50	50	1	-
M_30_PI_1_	30	70	1	-
M_90_PI_1_D_0.005_	90	10	1	0.005
M_90_PI_1_D_0.010_	90	10	1	0.010
M_90_PI_1_D_0.015_	90	10	1	0.015
M_80_PI_1_D_0.005_	80	20	1	0.005

**Table 2:** The compositions of the resins with bioactive glass investigated in this study.

Samples	m_BAG_/m_acr +_ m_BAG,_ [%]	m_TMPE,_ [%]	m_HEA_, [%]	m_PI_/m_acr,_ [%]	m_dye_/m_acr,_ [%]	m_PEG_/m_acr +_ m_PEG,_ [%]
20BM_90_PI_1_D_0.005_	20	90	10	1	0.005	-
30BM_90_PI_1_D_0.005_	30	90	10	1	0.005	-
40BM_90_PI_1_D_0.005_	40	90	10	1	0.005	-
50BM_90_PI_1_D_0.005_	50	90	10	1	0.005	-
60BM_90_PI_1_D_0.005_	60	90	10	1	0.005	-
60BM_90_PI_1_D_0.005_E_10_	60	90	10	1	0.005	10
60BM_90_PI_1_D_0.005_E_15_	60	90	10	1	0.005	15
45BM_90_PI_1_D_0.005_E_10_	45	90	10	1	0.005	10
55BM_90_PI_1_D_0.005_E_10_	55	90	10	1	0.005	10
55BM_90_PI_1_D_0.005_E_15_	55	90	10	1	0.005	15
65BM_90_PI_1_D_0.005_E_10_	65	90	10	1	0.005	10
55BM_80_PI_1_D_0.005_	55	80	20	1	0.005	-
55BM_80_PI_1_D_0.005_E_10_	55	80	20	1	0.005	10
40BM_80_PI_1_D_0.01_E_10_	40	80	20	1	0.01	10
55BM_80_PI_1_D_0.01_E_10_	55	80	20	1	0.01	10
60BM_80_PI_1_D_0.01_E_10_	60	80	20	1	0.01	10
55BM_80_PI_2_D_0.01_E_10_	55	80	20	2	0.01	10
55BM_80_PI_1_D_0.015_E_10_	55	80	20	1	0.015	10
60BM_80_PI_1_D_0.015_E_10_	60	80	20	1	0.015	10
50BM_90_PI_1_D_0_	50	90	10	1	-	-
50BM_90_PI_1_D*_0.01_*	50	90	10	1	0.01	

**Table 3:** Shore-D data of photocured samples (n = 5) in relation to PEG-200 content for compositions 55BM_90_PI_1_D_0.005_ and 60BM_90_PI_1_D_0.005_. Results are given in mean and standard deviation values.

Samples	55BM_90_PI_1_D_0.005_E_0_	55BM_90_PI_1_D_0.005_E_10_	55BM_90_PI_1_D_0.005_E_15_	60BM_90_PI_1_D_0.005_E_10_	60BM_90_PI_1_D_0.005_E_15_
Shore-D	71.4 ± 0.6	66.5 ± 0.4	61.4 ± 0.3	60.6 ± 0.5	57.1 ± 0.4

## Supplementary Materials

The following supporting information can be downloaded at: https://www.mdpi.com/article/10.3390/biomedicines10020395/s1, Table S1 (Properties of the applied acrylate monomers and polymers as given by suppliers), Table S2 (The compositions of the resins without BAG investigated in this study), Table S3 (The compositions of the bioactive glass containing resins investigated in this study) and Figure S1 (The SEM images of the 3D printed samples showing the absence of cracks).
