# Supplementary material for: Ceramic Stereolithography of Bioactive Glasses: Influence of Resin Composition on Curing Behavior and Green Body Properties
Source: Biomedicines. 2022 Feb 7;10(2):395. doi: 10.3390/biomedicines10020395 (PMC8962265; doi:10.3390/biomedicines10020395)
Supplement: Supplementary file 1 [file biomedicines-10-00395-s001.zip › biomedicines-1544469-supplementary.pdf]

Supporting Information

for

Ceramic Stereolithography of Bioactive Glasses: Influence of Resin Composition on Curing Behaviour and Green Body Properties

Qirong Chen[1*], Franziska Schmidt[2*], Oliver Görke[1‡], Anila Asif[3], Joachim Weinhold[4], Erfan Aghaei[1], Ihtesham ur Rehman[5], Aleksander Gurlo[1‡] and Asma Tufail Shah [1,3‡]

[1] Technische Universität Berlin, Institute of Materials Science and Technology, Fachgebiet Keramische Werkstoffe / Chair of Advanced Ceramic Materials, Hardenbergstr. 40, 10623 Berlin, Germany

[2] Dental Materials and Biomaterial Research, Department of Prosthodontics, Charite - Universitaetsmedizin Berlin, Aßmannshauser Str. 4–6, 14197 Berlin, Germany, email: Franziska.schmidt2@charite.de

[3] Interdisciplinary Research Centre in Biomedical Materials, COMSATS University Islamabad Lahore Campus, Defence Road, Off-Raiwand Road Lahore-54000, Pakistan

[4] Technische Universität Berlin, Faculty II - Mathematics and Natural Sciences, Institute of Mathematics, 3D Lab, Straße des 17. Juni 135, 10623 Berlin, Germany

[5] Engineering Department, Lancaster University, Gillow Ave, Bailrigg, Lancaster LA1 4YR, UK

* Both authors have contributed equally.

‡ Correspondence: OG o.goerke@tu-berlin.de, Tel.: +49 30 314-24976; ATS asma.tufail@campus.tu-berlin.de; AG gurlo@tu-berlin.de

Table S1. Properties of the applied acrylate monomers and polymers as given by suppliers. TMPE-OTA: Trimethylolpropane ethoxy triacrylate, HEA: 2-hydroxyethyl acrylate, PEG-200: Polyethylene glycol with molar mass 200 g/mol.

Material	Molar mass [g/mol]	Density [g/cm³]	Viscosity [mPa s]	Refractive Index [n20/D]
TMPE-OTA	268.3	1.11	50-75	1.471
HEA	116.1	1.011	11.17	1.450
PEG-200	190-210	1.124	60	1.460

Table S2 The compositions of the resins without BAG investigated in this study.

Samples	m_{TMPE} [g/%]	m_{HEA} [g/%]	PI, m and $m_{PI}/m_{acr,}$ [g/%]	Dye, m and $m_{dye}/m_{acr,}$ [g/%]
$M_{90}PI_1$	3.15/ 90	0.35/ 10	0.035 / 1	-
$M_{80}PI_1$	2.80/ 80	0.7/ 20	0.035 / 1	-
$M_{70}PI_1$	2.45/ 70	1.05/ 30	0.035 / 1	-
$M_{50}PI_1$	1.75/ 50	1.75/ 50	0.035 / 1	-
$M_{30}PI_1$	1.05/ 30	2.45/ 70	0.035 / 1	-
$M_{90}PI_1D_{0.005}$	3.15/ 90	0.35/ 10	0.035 / 1	0.000175/ 0.005
$M_{90}PI_1D_{0.010}$	3.15/ 90	0.35/ 10	0.035 / 1	0.00035/ 0.010
$M_{90}PI_1D_{0.015}$	3.15/ 90	0.35/ 10	0.035 / 1	0.000525/ 0.015
$M_{80}PI_1D_{0.005}$	2.80/ 80	0.7/ 20	0.035 / 1	0.000175/ 0.005

Table S3 The compositions of the bioactive glass containing resins investigated in this study.

Samples	$m_{BAG,}$ and $m_{BAG}/m_{acr}+m_{BAG,}$ [g/%]	m_{TMPE} [g/%]	m_{HEA} [g/%]	$m_{PI,}$ and $m_{PI}/m_{acr,}$ [g/%]	m_{dye}, and $m_{dye}/m_{acr,}$ [g/%]	$m_{PEG-200,}$ and $m_{PEG}/m_{acr}+m_{PEG,}$ [g/%]
$20BM_{90}PI_1D_{0.005}$	3/ 20	10.8/ 90	1.2/ 10	0.12 / 1	0.0006/ 0.005	-
$30BM_{90}PI_1D_{0.005}$	4.5/ 30	9.45/ 90	1.05/ 10	0.105 / 1	0.00052/ 0.005	-
$40BM_{90}PI_1D_{0.005}$	6/ 40	8.1/ 90	0.9/ 10	0.09 / 1	0.00045/ 0.005	-
$50BM_{90}PI_1D_{0.005}$	7.5/ 50	6.75/ 90	0.75/ 10	0.075 / 1	0.00037/ 0.005	-
$60BM_{90}PI_1D_{0.005}$	9/ 60	5.4/ 90	0.6/ 10	0.06 / 1	0.0003/ 0.005	-
$60BM_{90}PI_1D_{0.005}E_{10}$	9/ 60	4.86/ 90	0.54/ 10	0.054/ 1	0.00027/ 0.005	0.6/ 10
$60BM_{90}PI_1D_{0.005}E_{15}$	9/ 60	4.59/ 90	0.51/ 10	0.051/ 1	0.00026/ 0.005	0.9/ 15
$45BM_{90}PI_1D_{0.005}E_{10}$	6.75/ 45	6.68/ 90	0.74/ 10	0.074/ 1	0.0004/ 0.005	0.82/ 10
$55BM_{90}PI_1D_{0.005}E_{10}$	8.25/ 55	5.46/ 90	0.60/ 10	0.060/ 1	0.0003/ 0.005	0.67/ 10

$55BM_{90}PI_1D_{0.005}E_{15}$	8.25/ 55	5.16/ 90	0.57/ 10	0.057/ 1	0.0003/ 0.005	1.01/ 15
$65BM_{90}PI_1D_{0.005}E_{10}$	9.75/ 65	4.25/ 90	0.47/ 10	0.047/ 1	0.0002/ 0.005	0.25/ 10
$55BM_{80}PI_1D_{0.005}$	8.25/ 55	5.4/ 80	1.35/ 20	0.067/ 1	0.00034/ 0.005	-
$55BM_{80}PI_1D_{0.005}E_{10}$	8.25/ 55	4.86/ 80	1.215/ 20	0.060/ 1	0.00030/ 0.005	0.675/ 10
$40BM_{80}PI_1D_{0.01}E_{10}$	6/ 40	6.48/ 80	1.62/ 20	0.081/ 1	0.00081`/ 0.01	0.9/ 10
$55BM_{80}PI_1D_{0.01}E_{10}$	8.25/55	4.86/80	1.21/ 20	0.060/1	0.0060/ 0.01	0.675/ 10
$60BM_{80}PI_1D_{0.01}E_{10}$	9/ 60	4.32/ 80	1.08/20	0.054/ 1	0.00054/ 0.01	0.6/ 10
$55BM_{80}PI_2D_{0.01}E_{10}$	8.25/ 55	4.86/ 80	1.215/ 20	0.121/ 2	0.00060/ 0.01	0.675/ 10
$55BM_{80}PI_1D_{0.015}E_{10}$	8.25/ 55	4.86/ 80	1.215/ 20	0.06/ 1	0.00091/ 0.015	0.675/ 10
$60BM_{80}PI_1D_{0.015}E_{10}$	9/ 60	4.32/ 80	1.08/ 20	0.054/ 1	0.00081/ 0.015	0.60/ 10
$50BM_{90}PI_1D_0$	7.5/ 50	6.75/ 90	0.75/ 10	0.075 / 1	-	-
$50BM_{90}PI_1D_{0.01}$	7.5/ 50	6.75/ 90	0.75/ 10	0.075 / 1	0.00075/ 0.01	

Figures

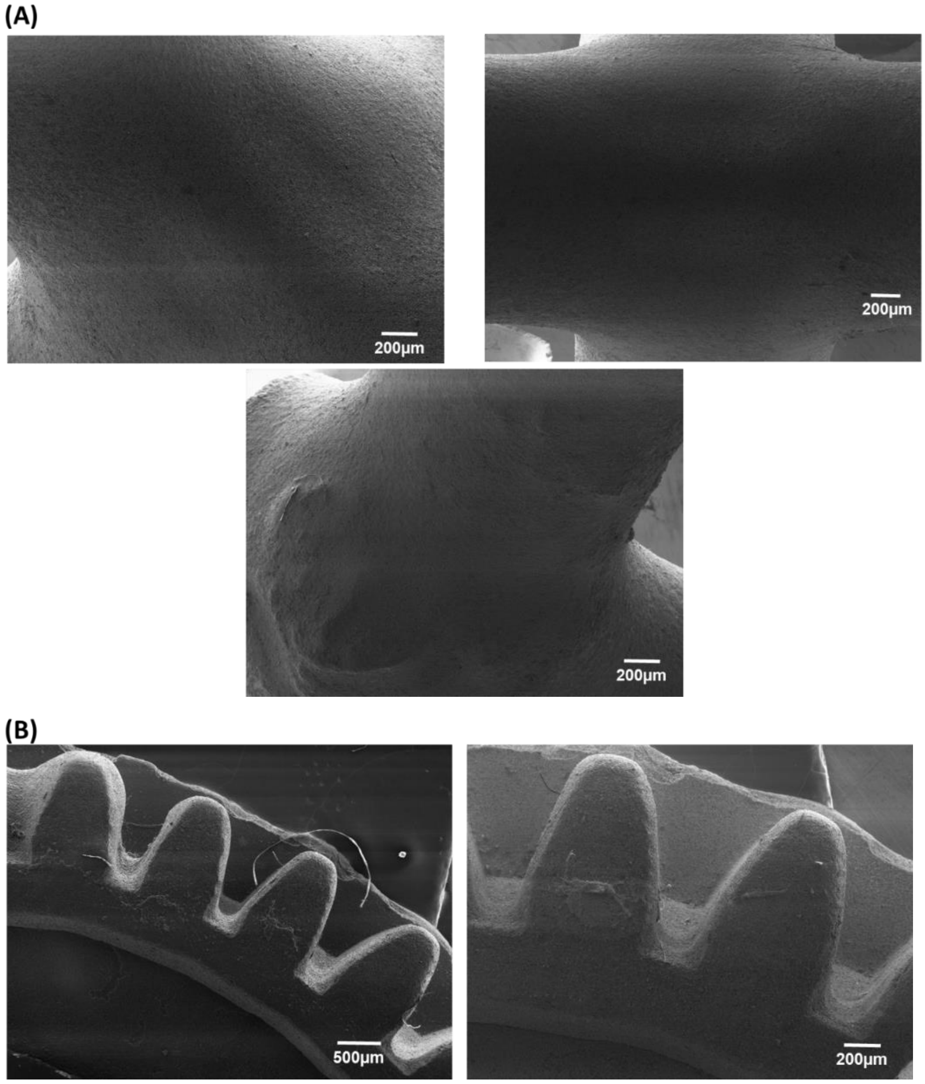

Figure S1 The SEM images of the 3D printed samples showing the absence of cracks, (A) 3D printed honey-comb structure (B) Gear-shaped object.
